# Peripheral Leukocyte Apoptosis in Patients with Parkinsonism: Correlation with Clinical Characteristics and Neuroimaging Findings

**DOI:** 10.1155/2014/635923

**Published:** 2014-03-26

**Authors:** Wei-Che Lin, Nai-Wen Tsai, Yung-Cheng Huang, Kuei-Yueh Cheng, Hsiu-Ling Chen, Shau-Hsuan Li, Chia-Te Kung, Yu-Jih Su, Wei-Ming Lin, Meng-Hsiang Chen, Tsui-Min Chiu, I-Hsiao Yang, Cheng-Hsien Lu

**Affiliations:** ^1^Department of Diagnostic Radiology, Kaohsiung Chang Gung Memorial Hospital, Chang Gung University College of Medicine, 123 Ta Pei Road, Niao Sung, Kaohsiung 83305, Taiwan; ^2^Department of Neurology, Kaohsiung Chang Gung Memorial Hospital, Chang Gung University College of Medicine, 123 Ta Pei Road, Niao Sung, Kaohsiung 83305, Taiwan; ^3^Department of Nuclear Medicine, Kaohsiung Chang Gung Memorial Hospital, Chang Gung University College of Medicine, 123 Ta Pei Road, Niao Sung, Kaohsiung 83305, Taiwan; ^4^Department of Internal Medicine, Kaohsiung Chang Gung Memorial Hospital, Chang Gung University College of Medicine, 123 Ta Pei Road, Niao Sung, Kaohsiung 83305, Taiwan; ^5^Department of Emergency Medicine, Kaohsiung Chang Gung Memorial Hospital, Chang Gung University College of Medicine, 123 Ta Pei Road, Niao Sung, Kaohsiung 83305, Taiwan; ^6^Department of Diagnostic Radiology, Chiayi Chang Gung Memorial Hospital, Chang Gung University College of Medicine, Chiayi, Taiwan

## Abstract

Apoptosis of both brain neurons and peripheral blood leukocyte is believed to be an important biomarker for evaluating the functional status of Parkinson's disease (PD). However, their correlation remains unknown. A better understanding of the pathophysiology of neurodegeneration is essential for the treatment and prevention of PD. The present study demonstrated that leukocyte apoptosis is significantly higher in PD patients and is associated with central dopamine neuron loss by using ^99m^Tc-TRODAT-1 SPECT. The leukocyte apoptosis and striatal dopamine transporter uptake ratios were further associated with increased severity and longer duration of disease. The interaction between brain and systemic inflammation may be responsible for the neurodegenerative disease progression.

## 1. Introduction

Parkinson's disease (PD) is a movement disorder caused by dopamine (DA) deficiency in the striatum due to DA neuron degeneration in the substantia nigra (SN). The etiopathogeny involves the interaction of environmental and genetic factors [1]. Recently, neuroinflammation has been considered fundamental to the progression of PD [2]. In postmortem analysis of PD patients, activated microglia is found in the SN pars compacta (SNpc) [3]. Elevated proinflammatory substances such as cyclooxygenase 2 (COX2) and cytokines including interleukin-1 beta (IL-1*β*), interferon-gamma (IFN-*γ*), and tumor necrosis factor alpha (TNF-*α*) are also found in postmortem PD brains [4–7], suggesting the presence of inflammatory processes [8].

Altered neurovascular functions in PD can lead to increased blood-brain barrier permeability and increased peripheral neutrophil and monocyte infiltration into the SN region, where they play an important role in neuroinflammation [9] and DA neuronal death. Recently, peripheral inflammation has been considered to have consequences on the degenerative process of DA neurons. In PD, some biochemical alterations affecting neuronal cells have been detected in circulating lymphocytes. Increased oxidative stress is associated with an imbalance between reactive oxygen species (ROS) formation and antioxidant defenses [10] and the presence of DNA damage [11]. Moreover, previous studies show a decreased number of circulating lymphocytes in PD patients [12]. Peripheral blood CD4+ T cells have increased susceptibility to apoptosis with Fas involvement in patients with PD [13]. Interestingly, some of these alterations may be associated with disease severity [14].

^99m^Tc-[2-[[2-[[[3-(4-chlorophenyl)-8-methyl-8-azabicyclo [3,2,1] oct-2-yl] methyl] (2-mercaptoethyl) amino]ethyl] amino]-ethanethiolato(3-)-N2,N2,S2, S2]oxo-[1R-(exo-exo)] (^99m^Tc-TRODAT-1) is a specific tracer developed to bind selectively to dopamine transporters in the brain. Studies with TRODAT-1 single photon emission computed tomography (SPECT) allow for an* in vivo* assessment of presynaptic dopaminergic neuron activity of the brain. ^99m^Tc-TRODAT-1 SPECT is a useful tool for differentiating Parkinsonian disorders [15]. Decreased striatal tracer uptake, indicating loss of DA neurons, can be used to evaluate worsening disease and confirm symptomatic lesions in the early stage of PD [16].

Apoptosis of both brain neurons and peripheral blood leukocyte is believed to be an important biomarker for evaluating the functional status of PD. However, their correlation remains unknown. Better understanding of the pathophysiology of neurodegeneration is essential for the treatment and prevention of PD. The present study hypothesized that leukocyte apoptosis plays an important role in the prognosis of PD. We analyzed the correlations among the peripheral leukocytes apoptosis, striatal neuronal loss on ^99m^Tc-TRODAT-1 SPECT/CT studies, and clinical presentations.

## 2. Materials and Methods

### 2.1. Subjects

Fifty-five PD patients (22 males, mean age 59.9 ± 10.9 years), without a history of other neurologic or psychiatric illness and psychotropic medications, contraindications to Madopar (L-dopa), at the Neurology Department of Chang Gung Memorial Hospital were prospectively enrolled. Patients were included when they had idiopathic PD, diagnosed according to the United Kingdom Brain Bank criteria [17] by an experienced neurology specialist. The time point of the diagnosis of PD was collected from each case, as well as the duration of disease. Disease onset was defined as the time of first recalled motor symptoms, such as tremor, bradykinesia, and rigidity in the pretreatment phase of the disease. Twelve patients never used any anti-Parkinson's medication, while the rest used dopaminergic medication (levodopa and dopamine agonists).

The studies were performed at least 12 h after the last dose of dopaminergic medication (off state). Each patient's disease severity and functional status were evaluated using the Unified Parkinson's Disease Rating Scale (UPDRS), the modified Hoehn and Yahr Staging Scale, and the Schwab and England Activities of Daily Living Scale. The Unified Parkinson's Disease Rating Scale (UPDRS) is the most commonly used scale to follow the longitudinal course of PD [18]. The UPDRS scores are evaluated by interview and clinical observation. The modified Hoehn and Yahr Scale provides a global assessment of severity in Parkinson's disease based on clinical findings and functional disability [19]. It is a commonly used system for describing how the symptoms of Parkinson's disease progress. It is a rating scale measured in an ordinal level and included stages 1 through 5. The higher rates describe an increased severity of the disease. The Schwab and England Activities of Daily Living Scale estimates the abilities of PD patients relative to a completely independent situation. One hundred percent indicates a completely independent patient and 0% indicates an individual in whom vegetative functions are no longer functioning [20].

For comparison, 37 sex- and age-matched healthy subjects (18 males; 62.9 ± 6.3 years) without a medical history of neurologic disease or psychiatric illness, alcohol or substance abuse, or head injury and with similar levels of education were recruited from the hospital. The hospital's Institutional Review Committee on Human Research approved the study and all of the participants or their guardians provided written informed consent.

### 2.2. Blood Sampling and Assessment of Leukocyte Apoptosis

Blood samples were collected from patients by venipuncture of forearm veins and from the control group upon enrollment. Blood sample analysis was done according to a previous work [21]. Whole blood (100 *μ*L) was stained with 10 *μ*L CD45-phycoerythrin- (PE-) Cy5 (clone J33) for 15 min at room temperature protected from light. The CD45-PE-Cy5 antibody reacts with the CD45 family of transmembrane glycoproteins, expressed on the surface of all human leukocytes, and is a pan-leukocyte marker. Cells were fixed with 5.5% formaldehyde. After washing, permeability was induced with permeability agent (Beckman Coulter) and the remaining erythrocytes were lysed. In this stage, the cells were brought into contact with APO 2.7-PE (clone 2.7A6A3; Immunotech, Marseille, France) for intracellular antigenic determinants. The APO 2.7-PE antibody reacts with the 38-kDa mitochondrial membrane protein (7A6 antigen), which is detectable on nonpermeabilized cells in the late apoptotic state [11]. Mouse immunoglobulin G-PE was used as a control for nonspecific staining. The leukocytes were then analyzed by flow cytometry.

Flow cytometry analysis was performed immediately after staining with an Epics XL flow cytometer (Beckman Coulter, Fullerton, CA) using the EXPO32 ADC software. Five thousand CD45-PE-Cy5+ cells per sample were acquired in combined forward and side scatters and deep-red FL4 fluorescence (CD45-PE-Cy5) leukocyte gate. Leukocyte subtypes were identified according to their CD45 expression intensity. The results were expressed as the percentage of specific fluorescence-positive cells. Apoptotic cells were defined by APO 2.7 positivity. A database coordinator was responsible for monitoring all data collection and entry. All data were checked for any inconsistencies. Intra-assay variability based on repeated measurements of the same blood sample was low.

### 2.3. ^99m^Tc-TRODAT-1 SPECT/CT and Region of Interest (ROI) Analysis

Each patient with PD was intravenously injected with a single bolus of 925 MBq (25 mCi) of ^99m^Tc-TRODAT-1. The image acquisitions were performed after 4 h using a dual-head SPECT/CT equipped with low-energy high-resolution collimators (Symbia T, Siemens Medical Solutions, Erlangen, Germany). Emission data were acquired in a 128 × 128 matrix with 1.45 zoom through 360° rotation (180° for each head) at 3° intervals for 30 s per angle step. Transmission data acquired by low-dose CT without contrast medium were used for attenuation correction and functional-anatomic image fusion.

**Figure 1:**
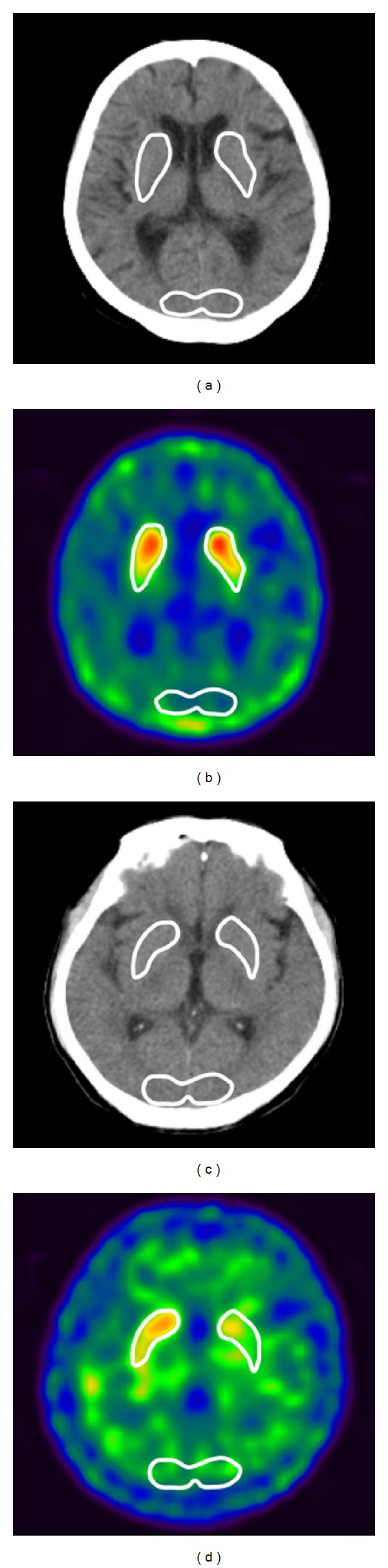
The ROIs of the striatal and occipital cortices (background) on ^99m^Tc-TRODAT-1 SPECT/CT imaging in patients with (a-b) normal and (c-d) abnormal striatal dopamine transporter uptake. The ROIs of the bilateral striatal and occipital cortices were drawn on the anatomic coregistration CT images (a and c) and transferred them to the transaxial composite slices of ^99m^Tc-TRODAT-1 SPECT images (b and d). The ROIs' radioactivities were counted for striatal dopamine transporter uptake analysis.

Low-dose CT images were acquired using the following parameters: 130 kV, 45 mAs (maximum), and 5 mm thick sections. Reconstruction and display of functional-anatomic fusion images were performed on the Syngo MI workplace (Siemens Healthcare, Forchheim, Germany). After FLASH 3D (ordered-subset expectation maximization iterative reconstruction method with 3D collimator beam modeling) reconstruction of the emission data, three-dimensional images of transaxial, coronal, and sagittal slices were obtained. The transaxial images of ^99m^Tc-TRODAT-1 SPECT/CT were analyzed both visually and semiquantitatively. With the help of anatomical coregistration CT images, ROIs of bilateral striata (including their subregions of caudate and putamen) were defined on composite images of the six highest striatum activity slices. The occipital cortex was drawn in the same way and served as background area (Figure 1). The ROIs' radioactivities were counted and striatal dopamine transporter uptake ratios were calculated as the quotient of the mean counts per pixel in each striatum divided by the mean counts per pixel in the occipital cortex. All images were reviewed by an experienced nuclear physician who was blinded to the patient's information.

### 2.4. Statistical Analysis

Data were expressed as mean ± SD or median (interquartile range). Univariate analyses used the Student's *t*-test or the Mann-Whitney test. For categorical variables, the *χ* ^2^ test or Fisher's exact test was used as appropriate. Partial Pearson's correlation analysis was used to explore the relationship between leukocyte apoptosis, clinical variables, and dopamine transporter uptake ratios in the PD group, after controlling for age and sex. Statistical significance was set at *P* < 0.05. All statistical analyses were performed using the SPSS software, version 10.0 (SPSS Inc., Chicago, IL).

## 3. Results

### 3.1. Baseline Characteristics of the Study Patients

The baseline characteristics and laboratory data of both groups (Table 1) showed no significant differences in age, sex, white blood cells, red blood cells, and platelet counts.

### 3.2. Leukocyte Apoptosis in the PD and Control Groups

**Figure 2:**
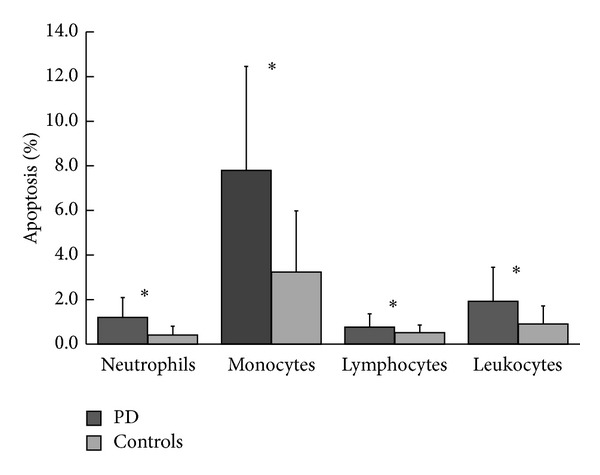
Apoptosis of total leukocytes and their subsets in PD patients and controls. *P < 0.001, PD patients versus controls.

The laboratory data, presented as medial value (interquartile range), and the percentage of leukocyte apoptosis of both groups (Figure 2) showed that the percentage of leukocyte apoptosis was significantly higher in PD patients than in controls (1.53 [1.03, 2.17] versus 0.81 [0.57, 1.17], *P* < 0.001). The percentages of apoptosis in the subsets of leukocytes, including neutrophils (0.89 [0.52, 1.37] versus 0.39 [0.24, 0.51], *P* < 0.001), monocytes (4.67 [3.22, 7.45] versus 2.74 [1.73, 4.27], *P* < 0.001), and lymphocytes (0.60 [0.47, 1.00] versus 0.36 [0.21, 0.68], *P* < 0.001), were significantly higher in the PD group (*P* < 0.01) than in controls.

### 3.3. Correlations of Percentage of Leukocyte Apoptosis, Striatal Dopamine Transporter Uptake Ratios, and Disease Severity of PD

The total leukocyte apoptosis negatively correlated with right striatal dopamine transporter uptake ratio (*γ* = −0.384, *P* = 0.012) and duration of disease (*γ* = −0.293, *P* = 0.039). The total leukocyte apoptosis and subset of neutrophil apoptosis positively correlated with disease severity in the UPDRS score and the modified H & Y score and negatively correlated with the S & E score. By further analysis, bilateral striatal dopamine transporter uptake ratios were negatively associated with UPDRS II, UPDR III, UPDR total scores, and the modified H & Y score and positively correlated with S & E score (Table 2).

## 4. Discussions

In the current study, changes in the number and composition of leukocyte subsets in PD suggested enhanced activation of apoptosis, which is consistent with previous findings [13]. The similar results have been extended by showing that the increase in apoptotic leukocytes is associated with striatal dopamine transporter uptake, suggesting a loss of DA neurons compared to healthy individuals. The relationship between leukocyte apoptosis and disease severity explains the increase in the amount of leukocyte subset apoptosis observed in Parkinson's disease and can be associated with the neurodegenerative process.

Apoptotic cell death occurs primarily through three different pathways: the extrinsic death receptor pathway (type I cells), the intrinsic (mitochondrial) pathway (type II cells), and the endoplasmic reticulum (ER) or stress-induced pathway [22]. All of these processes are accomplished or composed by different inflammatory processes. In the present study, higher leukocyte apoptosis level indicates higher peripheral inflammation in PD.

It is important to know whether peripheral inflammation, a very common health problem, can affect the degeneration of nigrostriatal dopaminergic neurons. Studies show that peripheral inflammation can enhance the degeneration of the nigrostriatal dopaminergic system. Systemic IL-1 expression may exacerbate neurodegeneration by an increase in inflammation in SN, as evidenced by the great reactivation of microglia. Studies also suggest that increases in other inflammatory cytokines (i.e., IL-6, IL-2, TNF-*α*, and IFN-*γ*) are responsible for this effect [23]. Interestingly, such proinflammatory, immunomodulatory, and anti-inflammatory cytokine alterations involved in apoptosis and enhanced oxidative stress [11] may be related to disease severity [14]. The result here on the high apoptosis of total leukocytes and subset of neutrophils associated with UPDRS score and disease duration further confirms previous studies. All of these data suggest that the increase in inflammatory parameters in the periphery (blood) due to peripheral inflammation induces an increase in inflammation in SN and, consequently, a synergistic effect on the nigrostriatal dopaminergic system.

^99m^Tc-TRODAT-1 shows promise as a tracer for the imaging of dopamine transporter [24], which is heavily expressed in the terminals of dopamine neurons that are lost in PD. The specific striatal binding of ^99m^Tc-TRODAT-1 shows a moderately negative correlation with disease severity and duration. This finding is similar to that of a previous study [16] and suggests that ^99m^Tc-TRODAT-1 is a useful marker of disease severity in PD, with potential utility for serial monitoring of disease progression. The decrease in striatal dopamine transporter uptake ratio suggests a loss of DA neurons in the substantia nigra.

Further exploring the relationship with the decrease in striatal dopamine transporter uptake ratio reveals a correlation in the increase in peripheral blood total leukocyte apoptosis and a weak correlation in the neutrophil subset. This may be due to the increase of oxidative stress in circulating leukocyte, especially in neutrophils [13], indicating that peripheral inflammation is correlated with dopamine dysfunction/cell loss. The level of neutrophils apoptosis further reflects the disease severity of PD. These findings suggest that circulation neutrophils apoptosis is a useful biomarker to assess disease status, and leukocytes apoptosis may play an important role in the pathogenesis of central neurodegeneration in PD.

Alteration of peripheral T-lymphocyte populations that increase susceptibility to apoptosis in PD has also been demonstrated [25]. In addition, disruption of the BBB with active lymphocyte infiltration to the brain induces inflammation in SN, such as microglial activation and increased proinflammatory cytokines [26]. However, the present results demonstrate no significant correlation between elevated monocyte and lymphocyte apoptosis and between disease severity and duration and striatal dopamine transporter uptake ratio. Immune reactions associated with cell death in SN are hypothesized to occur several years before the onset of symptomatic PD [27] and a series of peripheral immune alterations may precede the occurrence of immune reaction-related inflammation in the brain. Moreover, lymphocyte proliferation and cytokine production have been proposed to be affected by peripheral dopamine [28] such that levodopa therapy may also have a role in the alteration of lymphocytes populations in PD [29]. The pathophysiology of leukocyte profile alteration in PD is still unclear and further study of the peripheral leukocyte status in preclinical PD and its longitudinal evolution is warranted.

In conclusion, leukocyte apoptosis is significantly high in patients with PD and is associated with decreased striatal dopamine transporter uptake ratio implying central dopamine neuron loss. The interaction between brain and systemic inflammation may be responsible for the progression of this neurodegenerative disease. Investigating the relationship between the central and peripheral nervous system may help find targets for therapeutic interventions.

## Figures and Tables

**Table 1:** Demographic data of patients with PD and controls.

Clinical demographics	PD (*n* = 55)	Control (*n* = 37)	*P*
Age (year) (mean ± SD)	59.9 ± 10.9	62.9 ± 6.3	0.136
Sex (M, F)	22, 33	18, 19	0.089
White blood cells (×10^3^/mL)^#^	5.60 (5.00, 6.80)	6.05 (4.88, 8.43)	0.250
Red blood cells (×10^4^/mL)^#^	4.78 (4.42, 5.08)	4.41 (4.09, 5.00)	0.410
Platelet counts (×10^4^/mL)^#^	232 (200, 306)	223 (173, 276)	0.156
Duration of disease (years)^#^	2.5 (1.0, 5.5)		
UPDRS I^#^	3.0 (1.0, 6.0)		
UPDRS II^#^	10.0 (4.0, 16.0)		
UPDRS III^#^	22.0 (14.0, 34.0)		
UPDRS total^#^	33.0 (20.0, 54.0)		
Modified H & Y^#^ (maximum stage is 5)	1.75 (1.0, 3.0)		
S & E^# ^(minimum point is 0 suggesting vegetative functions)	90.0 (77.5, 100.0)		
TRODATE R^#^	1.45 (1.24, 1.62)		
TRODATE L^#^	1.37 (1.23, 1.53)		

UPDRS: Unified Parkinson's Disease Rating Scale; modified H & Y: modified Hoehn and Yahr Staging Scale; S & E: Schwab and England Activities of Daily Living Scale.

^#^Median (IQR): IQR: interquartile range.

**Table 2:** Correlation analysis between leukocyte apoptosis, ^99m^Tc-TRODAT-1 striatal uptake binding ratio, and clinical variables in the PD group after controlling for age and sex.

	Variables	*r*	*P* value
Total leukocyte apoptosis (%)	Striatal dopamine transporter uptake ratios mean	−0.349	**0.020**
	Striatal dopamine transporter uptake ratios *R*	−0.384	**0.012 **
	Striatal dopamine transporter uptake ratios *L*	−0.252	0.108
	UPDRS I	0.293	**0.041 **
	UPDRS II	0.480	**0.000 **
	UPDRS III	0.555	**0.000 **
	UPDRS total	0.537	**0.000 **
	Modified Hoehn-Yahr Staging Scale	0.461	**0.001 **
	Schwab and England Activities of Daily Living Scale	−0.463	**0.001 **
	Duration of disease	0.293	**0.039**

Neutrophil apoptosis (%)	Striatal dopamine transporter uptake ratios mean	−0.253	0.098
	Striatal dopamine transporter uptake ratios *R*	−0.272	0.082
	Striatal dopamine transporter uptake ratios *L*	−0.244	0.120
	UPDRS I	0.120	0.413
	UPDRS II	0.298	**0.037 **
	UPDRS III	0.356	**0.012 **
	UPDRS total	0.335	**0.019 **
	Modified Hoehn-Yahr Staging Scale	0.378	**0.007 **
	Schwab and England Activities of Daily Living Scale	−0.318	**0.026 **
	Duration of disease	0.145	0.315

Striatal dopamine transporter uptake ratios *R*	UPDRS III	−0.349	0.019
	UPDRS total	−0.330	0.027
	Modified Hoehn-Yahr Staging Scale	−0.373	0.012

Striatal dopamine transporter uptake ratios *L*	UPDRS II	−0.295	0.049
	UPDRS III	−0.328	0.028
	UPDRS total	−0.307	0.040
	Modified Hoehn-Yahr Staging Scale	−0.390	0.008
	Schwab and England Activities of Daily Living Scale	0.332	0.026

UPDRS: Unified Parkinson's Disease Rating Scale.
